# Supplementary material for: Seminoma and Embryonal Carcinoma Footprints Identified by Analysis of Integrated Genome-Wide Epigenetic and Expression Profiles of Germ Cell Cancer Cell Lines
Source: PLoS One. 2014 Jun 2;9(6):e98330. doi: 10.1371/journal.pone.0098330 (PMC4041891; doi:10.1371/journal.pone.0098330)
Supplement: File S1 — ZIP file containing DMRforPairs output for significant regions. Please start from the html files. (ZIP) [file pone.0098330.s008.zip › figures/13228.pdf]

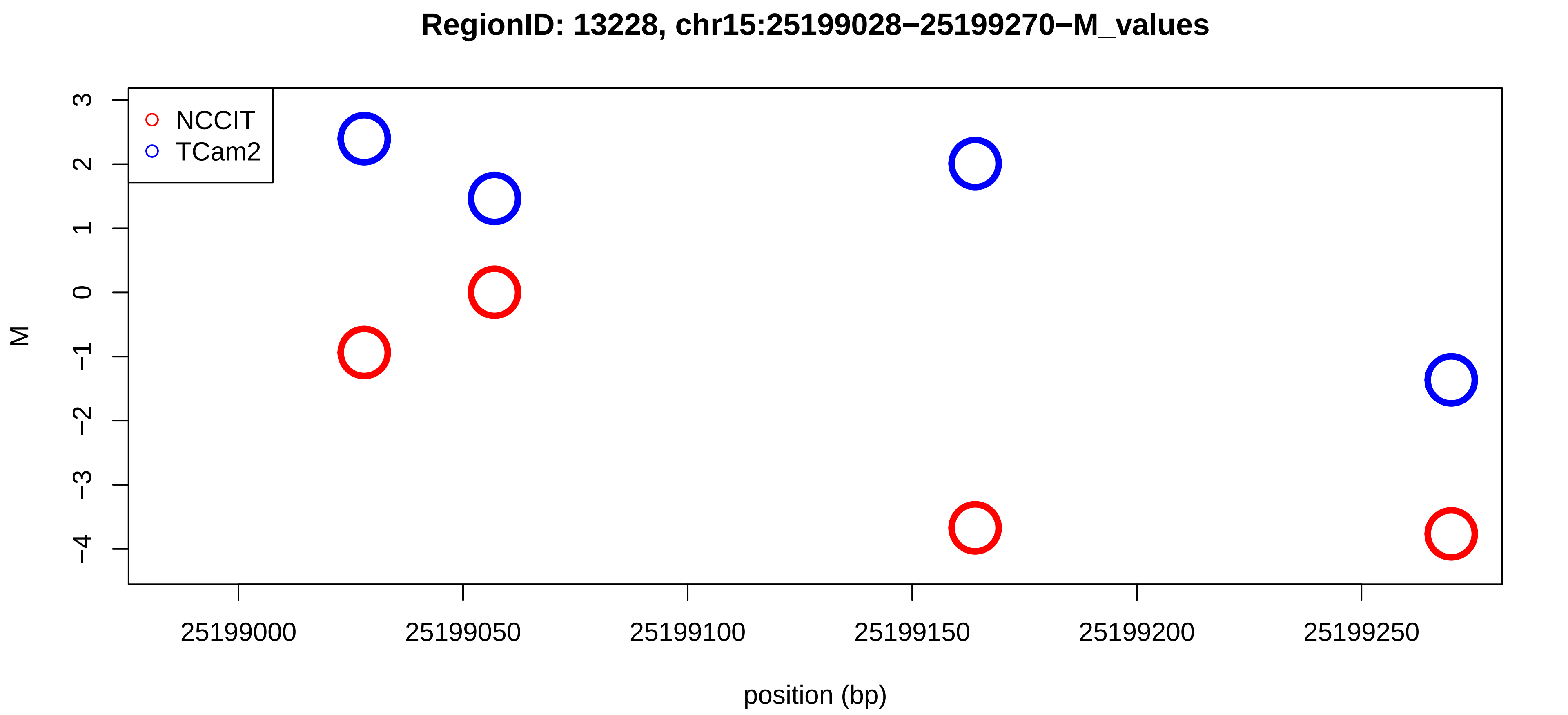

RegionID: 13228, chr15:25199028−25199270−M_values
NCCIT
TCam2
M
3
2
1
0
−1
−2
−3
−4
25199000
25199050
25199100
25199150
25199200
25199250
position (bp)

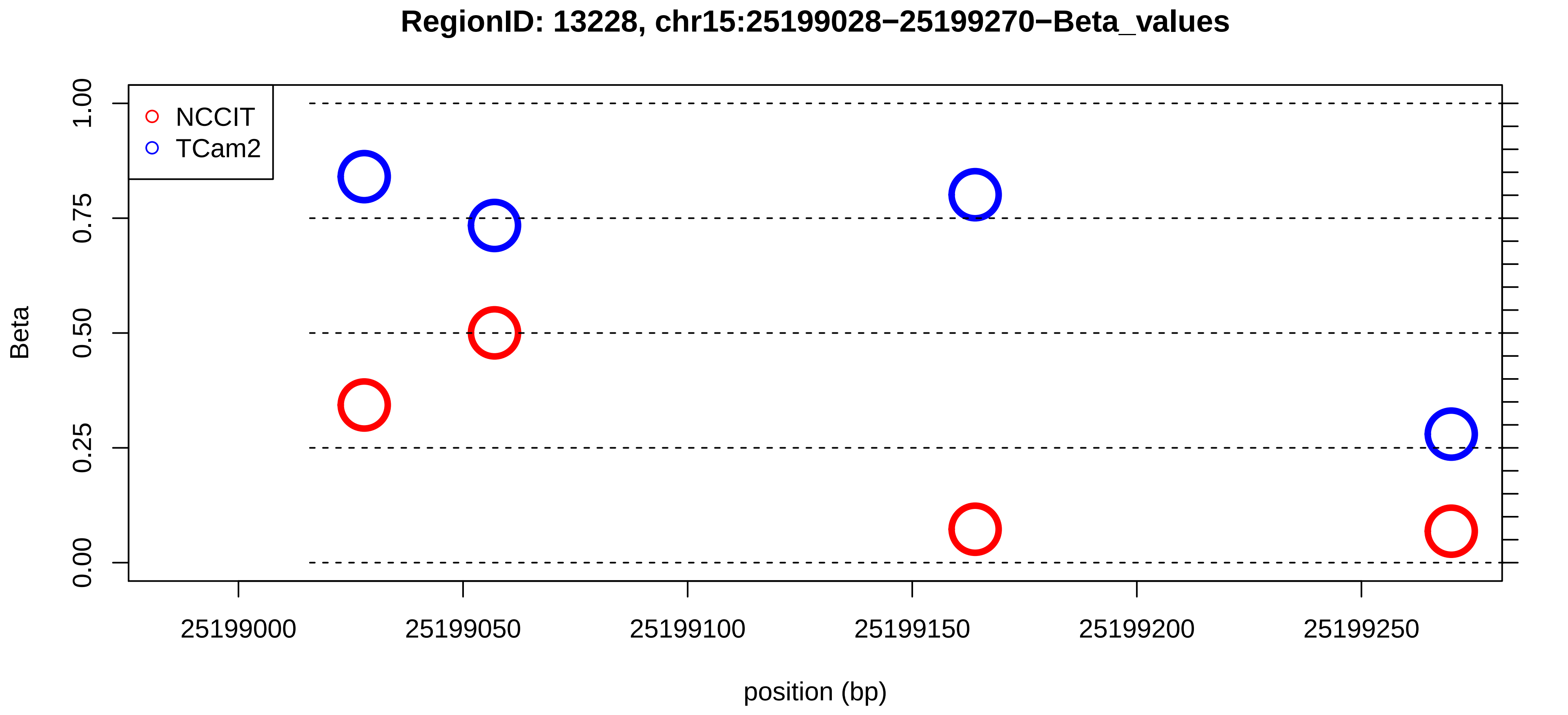

RegionID: 13228, chr15:25199028−25199270−Beta_values
NCCIT
TCam2
Beta
1.00
0.75
0.50
0.25
0.00
25199000
25199050
25199100
25199150
25199200
25199250
position (bp)
